# Comprehensive analysis of disruption mitigation methods using gas and pellet-like injections in ITER-like Tokamaks

**DOI:** 10.1038/s41598-025-14407-z

**Published:** 2025-09-22

**Authors:** V. Sizyuk, A. Hassanein

**Affiliations:** Center for Materials under Extreme Environment (CMUXE), Purdue University, West Lafayette, IN 47907 USA

**Keywords:** Magnetically confined plasmas, Computational methods, Fluid dynamics

## Abstract

Inert-gas shielding could be an effective mechanism for protection of plasma facing surfaces (PFS) against plasma particles impact and photon radiation heat loads during transient events in fusion devices. Neutral gas injection is one promising way to mitigate erosion of tokamak components and contamination. The objective of this work is to study and optimize mitigation methods using neutral gas and pellet-like injections to decrease the heat load to the divertor surfaces and to prevent vaporization of the various internal surfaces due to transient events in ITER-like devices. The integrated self-consistent models implemented in the HEIGHTS package was used for detailed analysis of the potential secondary plasma generation from the injected inert gas, its radiative characteristics, and shielding effectiveness. We varied the density, size, and location of an argon gas cloud to minimize the disruption energy deposited into the divertor components. We also investigated innovative ways for minor changes in ITER-like internal design to mitigate disruptions. We found the optimum parameters to fully protect ITER tokamak surfaces from erosion and vaporization during plasma instabilities. This preliminary analysis showed that using Ar gas injection methods could lead to enhancement in components lifetime in ITER-like and future DEMO devices with minor design changes.

**Supplementary Information:**

The online version contains supplementary material available at 10.1038/s41598-025-14407-z.

## Introduction

A successful development of thermonuclear fusion reactors based on the Tokamak concept such as ITER or next generation device DEMO is basically impossible without a robust solution to suppress or mitigate the heat load on plasma facing components (PFC) during transient events^1^. Currently there is no material capable to withstand the high heat and particle fluxes disrupting out of the hot core hydrogen isotope plasma to the divertor area during disruptions and edge localized modes (ELMs). This problem of PFC survivability remains a key concern even during the normal ITER tokamak operation where ELMs are considered regular expected events (~ 1 Hz)^2^. The intense energetic flux of particles escaping the hot core is deposited on a small area on the divertor plates near the separatrix. Most studies and experimental data of current machines still suggest disruptions will occur on the divertor plates around the strike point^3–5^. The predicted ITER disruptive plasma energy of 126 MJ during disruption will result in up to ~ 50 GW/m^2^ heat flux on the surface that vaporizes the divertor material at the strike point (SP) spot in ~ 1 µs after the start of the disrupted particles impact^6^. At the same time, the heat load limit on the surface of an actively cooled tungsten divertor is estimated to be not more than 10–15 MW/m^2^^7^.

The shielding of the vaporized divertor material can be an effective mechanism for the protection of the divertor surfaces against the charged particles and photon radiation. Formation of a secondary dense plasma cloud above the divertor surface leads to reduction of the heat flow to plasma facing material (PFM) at the strike point due to redirection of the impact energy deposition. The energy of the hot core particles is deposited into the vapor/plasma cloud resulting in heating and ionization of this cloud. In the case of unmitigated disruption or giant ELM in ITER with pure tungsten divertor, this secondary plasma is composed of high-Z tungsten ions. As a result, the energy flux of photons generated from such dense secondary plasma is comparable with the energy flux of the original core particles. The photon radiation from the secondary plasma cloud can however, cause significant damage to the divertor nearby components^8^. In contrast to high-Z materials, the photon radiation from low-Z plasma generated from a potential low-Z divertor plate is not very intense. However, low-Z plasma cloud can help dissipate the power of particle fluxes leading to cooling of the plasma near the divertor up to temperatures at which the plasma begins to recombine within the cloud before reaching any other surface. This is so called divertor detachment that is considered as the main operating mode of the ITER reactor, as well as future power plants^9^. The lifetime of plasma transient events is in the order of 1 ms. In this work, we calculated the required parameters of an injected inert gas cloud near the divertor plate to fully prevent any vaporization of the divertor and nearby components. Such injection should resemble an airbag with a very high speed release of the inert gas when sensing a disruption. Currently, different strategies and methods are used to predict disruptions in tokamaks^10^. We simulated here the perfect conditions to see even if possible that gas injection will offer sufficient protection during the predicted transient event.

Our previous studies showed that an insert of carbon strip at the SP in the tungsten divertor can significantly reduce photon radiation and the damage from the secondary divertor carbon plasma formed during unmitigated disruptions and ELMs^11^. However, using carbon as PFM has several disadvantages. First, carbon increases tritium accumulation during normal operation due to co-deposition with carbon. Second, while carbon insert leads to formation of secondary low-Z plasma during transient events which acts similar to low-Z gas injection, the main source of impurities in this case is from sputtering. The sputtering processes are difficult to control during the event. Also, the sputtering area is localized mainly at the SP. Development of effective detachment regimes requires more controllable and spatially movable influx of the low-Z gas. Sudden injection of low-Z gas (i.e., airbag like) into the divertor space is one of the most promising ways to mitigate damage and erosion of tokamak components. Typically, injection of low-Z impurities, e.g., nitrogen or neon, into the divertor region is considered in order to achieve the detachment regimes in tokamaks with metallic PFMs. Mitigation methods using gas injection involves variety of optimization parameters, depending on the tokamak power and energy fluxes. Parameters of injected gas, such as density, atomic number, injection location, etc. should be optimized in connection with the magnetic configuration and reactor design geometry to avoid the direct impact of the hot core plasma onto other components surface. The balance between energy of the escaped core plasma particles and photon radiation from the secondary generated divertor plasma should be optimized to decrease the local energy deposition and to prevent melting and vaporization of surrounding surfaces. If achievement of the detachment mode requires higher energy dissipation by photon radiation, higher-Z gas such as Ar, Kr, Xe may be injected. In addition to control the source of impurities in tokamak, the dynamics of the secondary plasma cloud and related parameters as temperature, density, and velocity distribution requires complex integrated analysis. When all details of gas parameters, magnetohydrodynamic (MHD), and radiation transport (RT) processes are taken into account in the evolving secondary plasma, mitigation by gas injection may be controlled.

The evolution of the secondary plasma cloud is dynamic due to the unsteady character of the energy deposition into the secondary plasma and the nonuniform heating of the cloud volume as it expands. In addition, the secondary dense plasma cloud itself acts as a scattering source for the incident transient core plasma particles. The scattered particles will be redirected and deposit their energy on various divertor and other component surfaces. The question arises, if it is possible to completely protect both the divertor surface during a disruption in ITER-like devices and to avoid any vaporization or melting areas/spots on the nearby components using neutral gas injection, pellet-like injection, or slight design modifications? The objective of this work is to answer this question and accurately evaluate the effectiveness of gas injection methods through comprehensive simulations of the core particles impact and interaction with injected neutral gas (Ar) and potential formation of a secondary plasma cloud of the gas and its evolution dynamics. This comprehensive analysis will help determine the optimum mitigation strategy during ITER-like disruption or ELM events. We varied the argon gas injection parameters such as density, size, and location in order to find conditions to avoid surfaces vaporization which should be more serious than just melting.

### Integrated model details

To perform this study, we upgraded the integrated physical models and HEIGHTS computer package^12^ and added capabilities to simulate transient events mitigation by neutral gas injections. We incorporated into our integrated model^13^ the MHD equations set for the injected gas/plasma dynamic simulations:

1 $$\:\begin{array}{c}\begin{array}{c}\begin{array}{c}\begin{array}{c}\begin{array}{c}\begin{array}{c}\begin{array}{c}\frac{\partial\:\rho\:}{\partial\:t}+\frac{1}{r}\frac{\partial\:}{\partial\:r}\left(r\rho\:{v}_{r}\right)+\frac{\partial\:}{\partial\:z}\left(\rho\:{v}_{z}\right)=0\\\:\frac{\partial\:\rho\:{v}_{r}}{\partial\:t}+\frac{1}{r}\frac{\partial\:}{\partial\:r}\left[r\left(\rho\:{v}_{r}^{2}-\frac{{B}_{r}^{2}}{4\pi\:}\right)\right]+\frac{\partial\:{p}_{t}}{\partial\:r}+\frac{\partial\:}{\partial\:z}\left(\rho\:{v}_{r}{v}_{z}-\frac{{B}_{r}{B}_{z}}{4\pi\:}\right)=\frac{\rho\:{v}_{\phi\:}^{2}}{r}-\frac{{B}_{\phi\:}^{2}}{4\pi\:r}\end{array}\\\:\frac{\partial\:\rho\:{v}_{\phi\:}}{\partial\:t}+\frac{1}{r}\frac{\partial\:}{\partial\:r}\left[r\left(\rho\:{v}_{\phi\:}{v}_{r}-\frac{{B}_{\phi\:}{B}_{r}}{4\pi\:}\right)\right]+\frac{\partial\:}{\partial\:z}\left(\rho\:{v}_{\phi\:}{v}_{z}-\frac{{B}_{\phi\:}{B}_{z}}{4\pi\:}\right)=\frac{\rho\:{v}_{\phi\:}{v}_{r}}{r}-\frac{{B}_{\phi\:}{B}_{r}}{4\pi\:r}\end{array}\\\:\frac{\partial\:\rho\:{v}_{z}}{\partial\:t}+\frac{1}{r}\frac{\partial\:}{\partial\:r}\left[r\left(\rho\:{v}_{z}{v}_{r}-\frac{{B}_{z}{B}_{r}}{4\pi\:}\right)\right]+\frac{\partial\:}{\partial\:z}\left(\rho\:{v}_{z}^{2}+{p}_{t}-\frac{{B}_{z}^{2}}{4\pi\:}\right)=0\end{array}\\\:\frac{\partial\:{e}_{h}}{\partial\:t}+\frac{1}{r}\frac{\partial\:}{\partial\:r}\left\{r\left[{v}_{r}\left({e}_{h}+{p}_{h}\right)\right]\right\}+\frac{\partial\:}{\partial\:z}\left[{v}_{z}\left({e}_{h}+{p}_{h}\right)\right]={Q}_{imp}+{Q}_{rad}+{Q}_{hc}+{Q}_{J}\end{array}\\\:\frac{\partial\:{B}_{r}}{\partial\:t}+\frac{\partial\:}{\partial\:z}\left({v}_{z}{B}_{r}-{B}_{z}{v}_{r}\right)={Q}_{r}^{md}\end{array}\\\:\frac{\partial\:{B}_{\phi\:}}{\partial\:t}+\frac{\partial\:}{\partial\:r}\left({v}_{r}{B}_{\phi\:}-{B}_{r}{v}_{\phi\:}\right)+\frac{\partial\:}{\partial\:z}\left({v}_{z}{B}_{\phi\:}-{B}_{z}{v}_{\phi\:}\right)={Q}_{\phi\:}^{md}\end{array}\\\:\frac{\partial\:{B}_{z}}{\partial\:t}+\frac{1}{r}\frac{\partial\:}{\partial\:r}\left[r\left({v}_{r}{B}_{z}-{B}_{r}{v}_{z}\right)\right]={Q}_{z}^{md}\end{array}$$

**Fig. 1:**
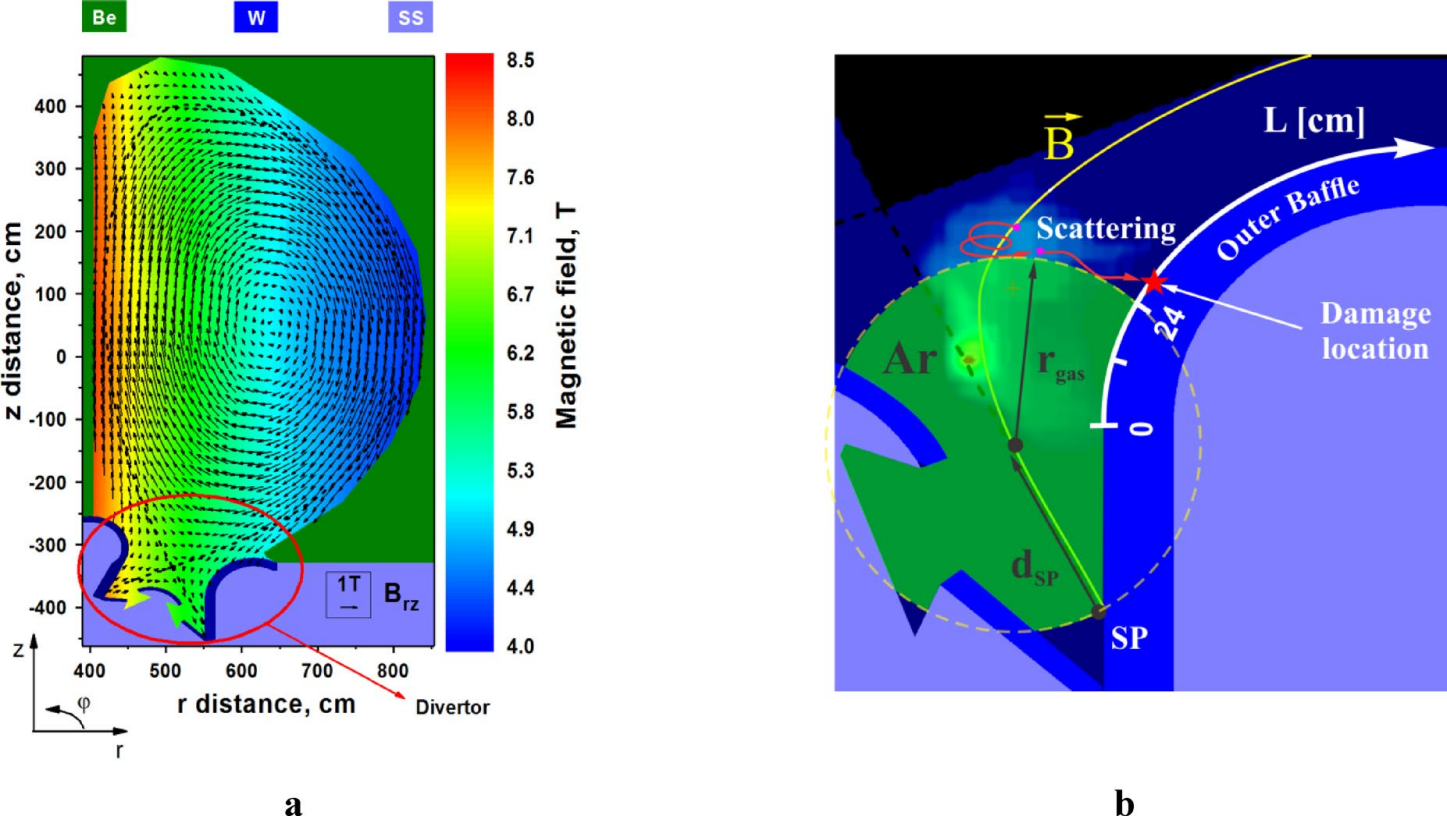
Schematic illustration of simulating ITER device: coordinate system orientation, initial magnetic field, separatrix **a**; and neutral gas injection into divertor space **b**. The images were prepared using OriginPro V2020.

Equation (1) describe the conservation laws for mass, momentum, energy, and magnetic field given in the cylindrical coordinate system (Fig. 1a). Here we assumed, *ρ* is the injected gas/plasma density; *v* is gas/plasma velocity; *B* is magnetic field; *p*_*t*_ *= p*_*h*_ *+ p*_*m*_ is total pressure including hydrodynamics and magnetic parts; *e*_*h*_ *= e*_*i*_ *+ e*_*k*_ is total hydrodynamic energy including internal and kinetic energy of gas/plasma. We took into account the details of the dissipative physical processes (core plasma energy deposition during the transient events, heat conduction, radiation transport, magnetic diffusion) as shown in the right-side sources in Eq. (1). The source terms in the right side include: *Q*_*hc*_ is the thermal conduction source, determine the solution of the heat conduction equation; *Q*_*rad*_ is radiation source, determine the solution of the RT block; *Q*_*J*_ is Joule heat term, determine the effect of Joule heating currents induced in plasma; *Q*_*imp*_ is core plasma source, determine the energy deposited by escaped core particles, also determine the solution of the MC plasma particles impact term; and *Q*^*md*^ is magnetic diffusion term, determine the solution of the resistive part of the magnetic field equations. Generally, the source terms are the solution results of the additional equations that describe the specific physical processes of the various sub-problems. The source terms adjust the transport model by taking into account the anisotropic transport properties that incorporates radial and toroidal solutions into Eq. (1). The descriptions of the source terms implementation were given in details in Refs^6,13–15^.

Figure 1a schematically shows the poloidal cross-section of ITER design, the coordinate system used, and the initial magnetic field configurations from the EQDSK files^16^. The following initial conditions were used in these simulations. The neutral gas Ar was injected into the divertor space as a toroidal cloud centered at the separatrix (see Fig. 1b), where $\:{d}_{SP}$ is the distance from the cloud center to SP and $\:{r}_{gas}$ is radius of the injected gas cloud. For convenience, we implemented an additional scale along the Baffle surface to monitor the heat load on the surfaces and to detect any potential vaporization spots. The origin of the surface scale is located at the end of the divertor plate border, marking the beginning of the Baffle surface.

The second key capability introduced into HEIGHTS is the possibility to modify the design of the interior components slightly shifting them from their original location. For example, in this study we used the outer Baffle shift along the z-axis as a parameter for the mitigation of the heat load distribution during the disruption. Thereby, the main input parameters were selected in this study for the optimization of the disruption energy distribution and prevention of vaporization spots: neutral gas Ar density, size and location of the gas cloud, and the outer Baffle vertical shift. The simulations were stopped in the case of any surface vaporization detected on any internal and nearby components surface. The start time of vaporization and the location of the damaged spots are the main output parameters tracked during these mitigation/optimization processes.

### Disruption dynamic simulation results

**Fig. 2:**
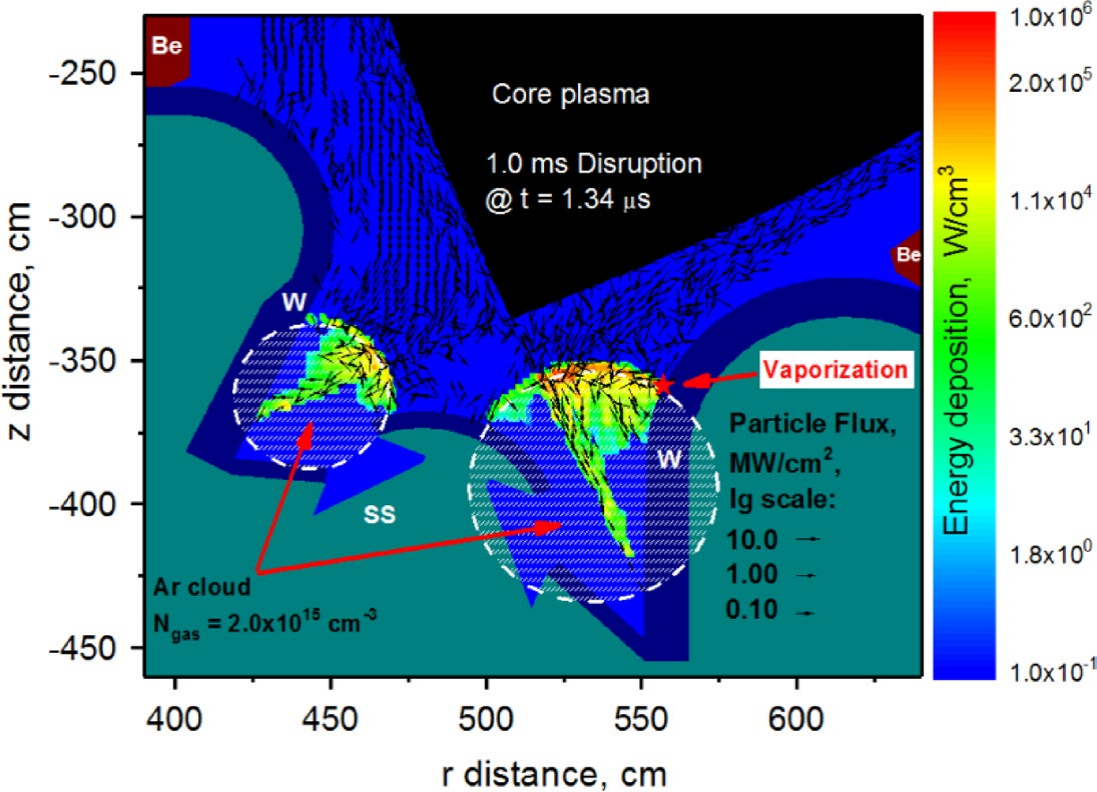
HEIGHTS simulation of the 1 ms disruption with Ar filled divertor space. The images were prepared using OriginPro V2020.

As in our previous studies^8,11,12^, we assumed for the 1 ms disruption the release of the full pedestal energy $\:{Q}_{DIS}$ = 126 MJ with the pedestal plasma temperature $\:{T}_{ped}$ = 3.5 keV. In our first simulation, the divertor space was fully filled with a cold $\:T$ = 500 K Ar gas as it is shown in Fig. 2 (dashed area). The outer divertor space was shielded with Ar gas cloud radius $\:{r}_{gas}$ = 40 cm and distance to SP $\:{d}_{SP}$ = 40 cm. This is to resemble an airbag triggered by an anticipated disruption event. The inner divertor space was also filled correspondingly $\:{r}_{gas}$ = 20 cm and $\:{d}_{SP}$ = 25 cm. The injected gas density $\:{N}_{gas}$ was varied in wide range from 1 × 10^15^ cm^−3^ up to 1 × 10^17^ cm^−3^.

**Fig. 3:**
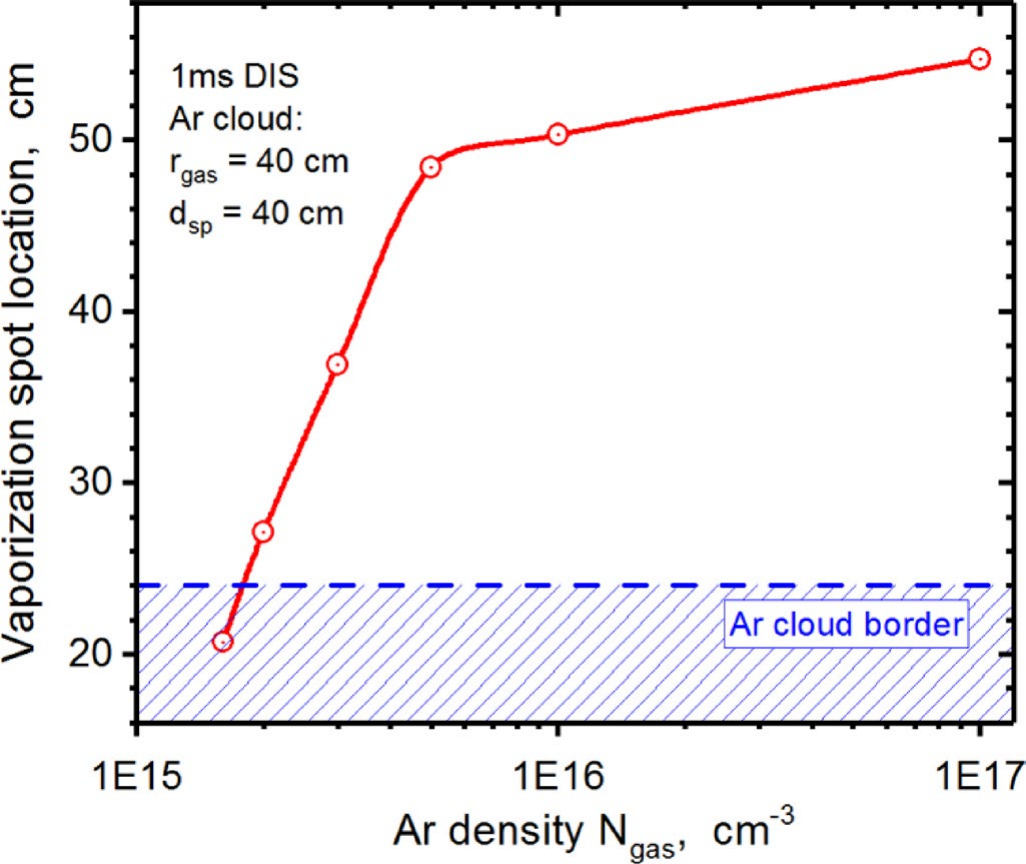
HEIGHTS simulation of the vaporization spot location during the 1 ms disruption vs. injected Ar gas density. The images were prepared using OriginPro V2020.

HEIGHTS dynamic simulations showed the presence of two scenarios of particle scattering and deposition sources: (1) direct penetration to the SP through the cloud when the gas density is small and (2) scattering on the gas cloud border with drift through the SOL to the unshielded Baffle surface in the case of higher density of the injected gas. Figure 2 clearly shows both ways for the Ar gas density $\:{N}_{gas}$ = 2.0 × 10^15^ cm^−3^ at the start of the 1.0 ms disruption input ($\:t$=1.34 µs). The first scenario leads to the SP vaporization of the divertor plate and the second one from plasma particles scattering that results in the vaporization of the Baffle surface. We did not find any intermediate Ar gas density which could lead to full protection from the vaporization of all components. The high-Z tungsten plasma formation occurred for all range of Ar gas densities. For the assessment of the Ar plasma shielding efficiency, we introduced two parameters: the vaporization spot location on the divertor surface $\:{L}_{vap}$ (see scale in Fig. 1b) and the start time of the vaporization $\:{t}_{vap}$ after the start of disruption. We found that the vaporization spot location smoothly shifts upwards from the SP to the Baffle as the Ar density increases as shown in Fig. 3. The vaporization spot leaves the initially Ar covered area (Ar cloud edge $\:{L}_{gas}$=24 cm) at the gas density $\:{N}_{gas}$ ~ 1.8 × 10^15^ cm^−3^. See please Fig. 1b for Baffle scale. The injected gas cloud is not an effective shield below this critical density. Above this value, the Ar cloud becomes a powerful reflector for the incoming plasma particles. At lower gas density, disrupting core plasma particles penetrate through the Ar gas and deposits most of their energy on the divertor plate around the strike point. Then at higher gas densities, most of the core plasma particles deposit their energy near the top of the expanding Ar plasma along the SOL along the Baffle as the initial Ar density increases. The slope in Fig. 3 changed according to these limits where the gas density was varied over a wide range.

**Fig. 4:**
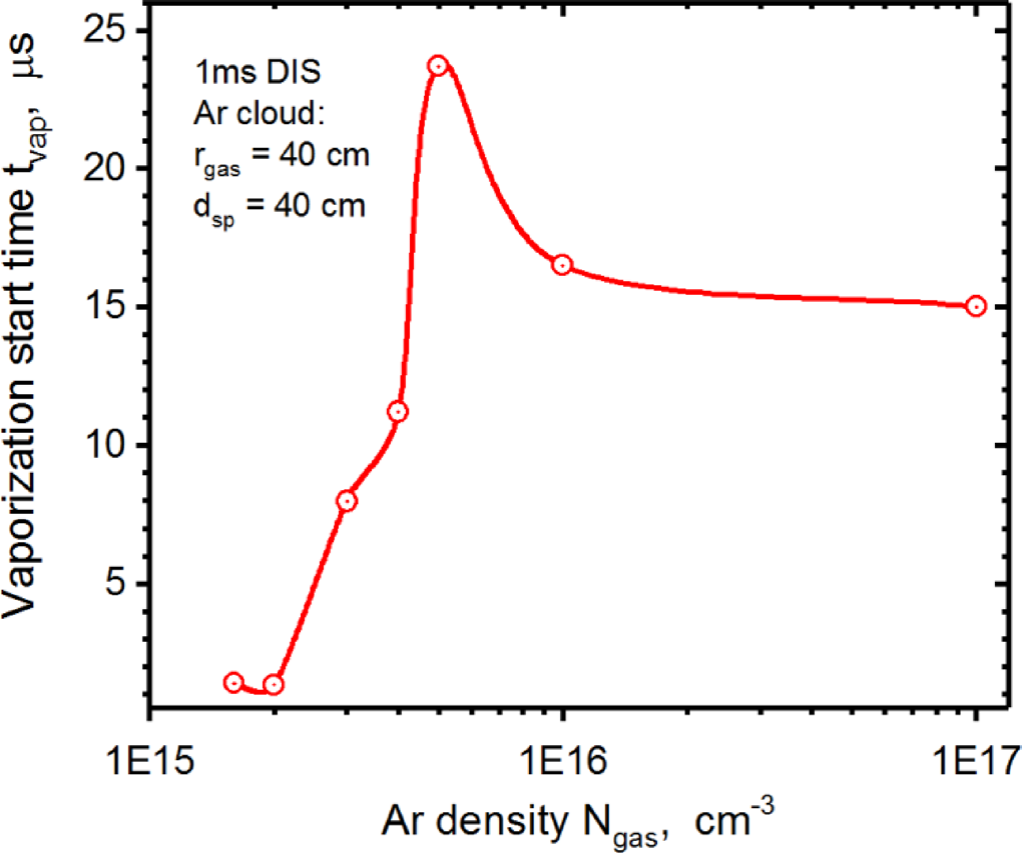
HEIGHTS simulation of the vaporization start time after 1 ms disruption vs. injected Ar gas density. The images were prepared using OriginPro V2020.

The time dependence of the vaporization on the injected gas density is not linear and has a peak at $\:{t}_{vap}$ = 23.7 µs for the $\:{N}_{gas}$ = 5.0 × 10^15^ cm^−3^. Taking into account that the vaporization time without Ar gas injection is about ~ 1 µs, surface damage can be delayed up to a maximum of ~ 23 µs when Ar gas injection is used to mitigate disruptions (Fig. 4). This vaporization delay peak can be explained by the competition of the two scenarios of particle scattering and deposition sources mentioned above: penetration through the Ar gas cloud and the scattering of the incident plasma particles on the cloud border.

**Fig. 5:**
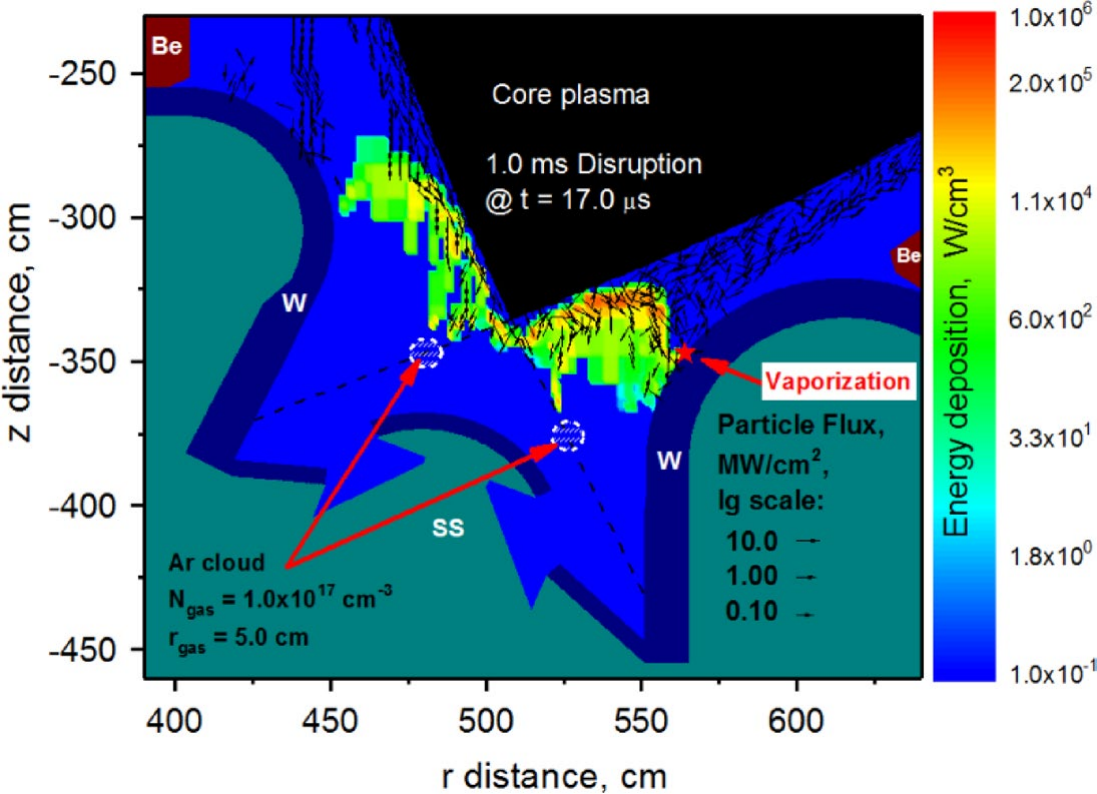
HEIGHTS simulation of the 1 ms disruption with the Ar cloud size and location variation. The images were prepared using OriginPro V2020.

The simulations showed that simple filling of the divertor space with Ar gas did not show a clear advantage and cannot be considered as an effective mitigation of surface damage during ITER-like disruption. Figure 5 shows the second mitigation method to prevent disruption damage. This method resembles localized gas injection, i.e., pellet-like injection. This localized Ar cloud was varied in size ($\:{r}_{gas}$) and in location ($\:{d}_{SP}$) within the divertor space. Preliminary calculations showed that variation of the gas density ($\:{N}_{gas}$) results in similar effect to the case of fully filled divertor space, i.e., resulting in two sources of particles scattering and deposition and do not show complete mitigation. Based on these estimations, the density of injected Ar gas was fixed at the $\:{N}_{gas}$ = 1.0 × 10^17^ cm^−3^ for the second mitigation method. We have also kept the output parameters the same, i.e., time to vaporization after the disruption starts and location of the damaged spots.

**Fig. 6:**
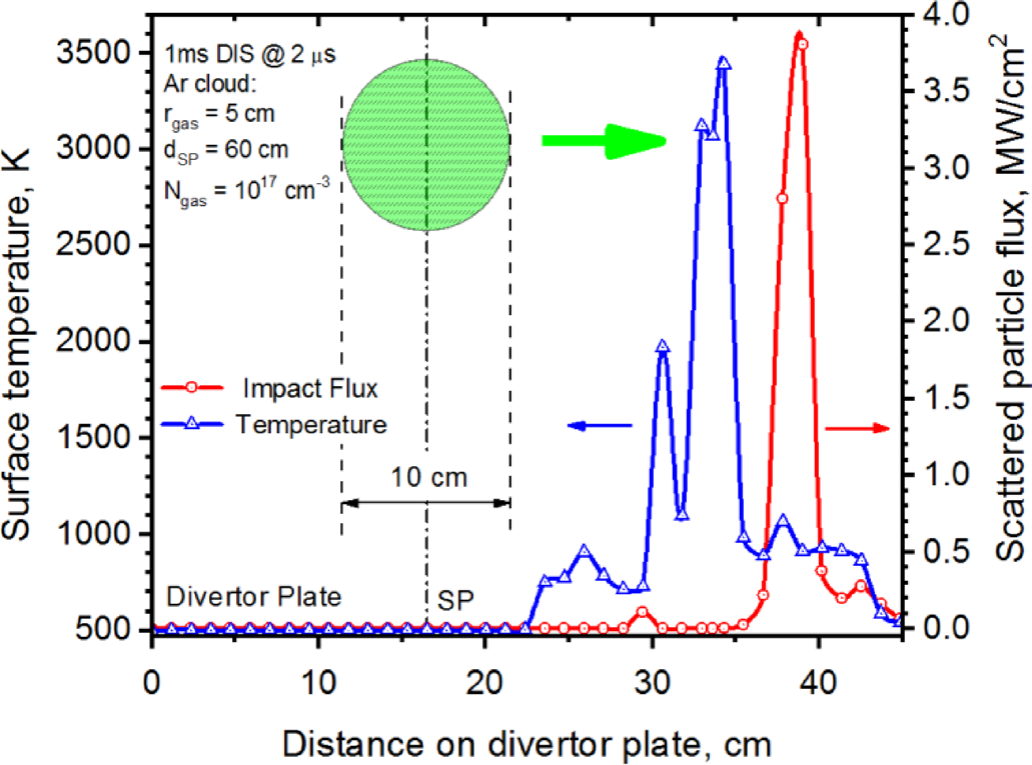
Shielding of the strike point by the localized Ar gas cloud at the separatrix. The curves move right with the disruption transition. The images were prepared using OriginPro V2020.

The localized Ar gas cloud near the separatrix is an effective scattering and shielding media where the core plasma particles energy flux (red curve) and surface temperature (blue curve) is shown in Fig. 6 at the early stages of the 1 ms disruption (t = 2 µs). Here, the scale is located on the divertor plate surface having the origin at the plate border along the Baffle scale (Fig. 1b). The SP is fully shielded by the Ar gas (r_gas_ = 5 cm) of density $\:{N}_{gas}$ = 1.0 × 10^17^ cm^−3^. In this case, the Ar cloud is heated by the incoming disruptive plasma particles and aggressively expanding. As a result, the peak scattered particles flux moves along the divertor plate surface (green arrow) during the disruption event. The peak of the impact flux (red curve) causes the surface temperature rise (blue curve) and causes the vaporization spot (red star) as shown in Fig. 5.

**Fig. 7:**
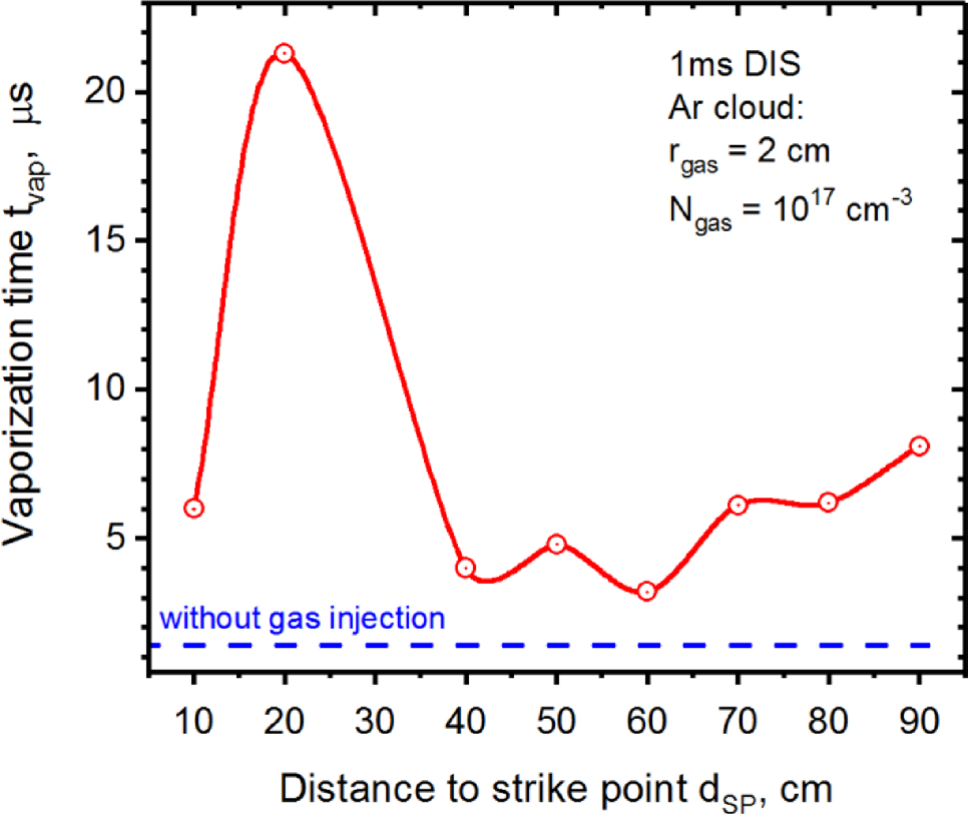
HEIGHTS simulation of the 1 ms disruption with variable Ar cloud (2 cm) location. The images were prepared using OriginPro V2020.

Variation of the small and localized Ar gas cloud ($\:{r}_{gas}$ = 2 cm), i.e., pellet-like injections along the separatrix results in similar behavior to the airbag-like mitigation method with a peak in 21.3 µs (Fig. 7). A full mitigation is also not found in this method. Vaporization of the Baffle surface at most was delayed for 20 µs after the start of the disruption in this case.

**Fig. 8:**
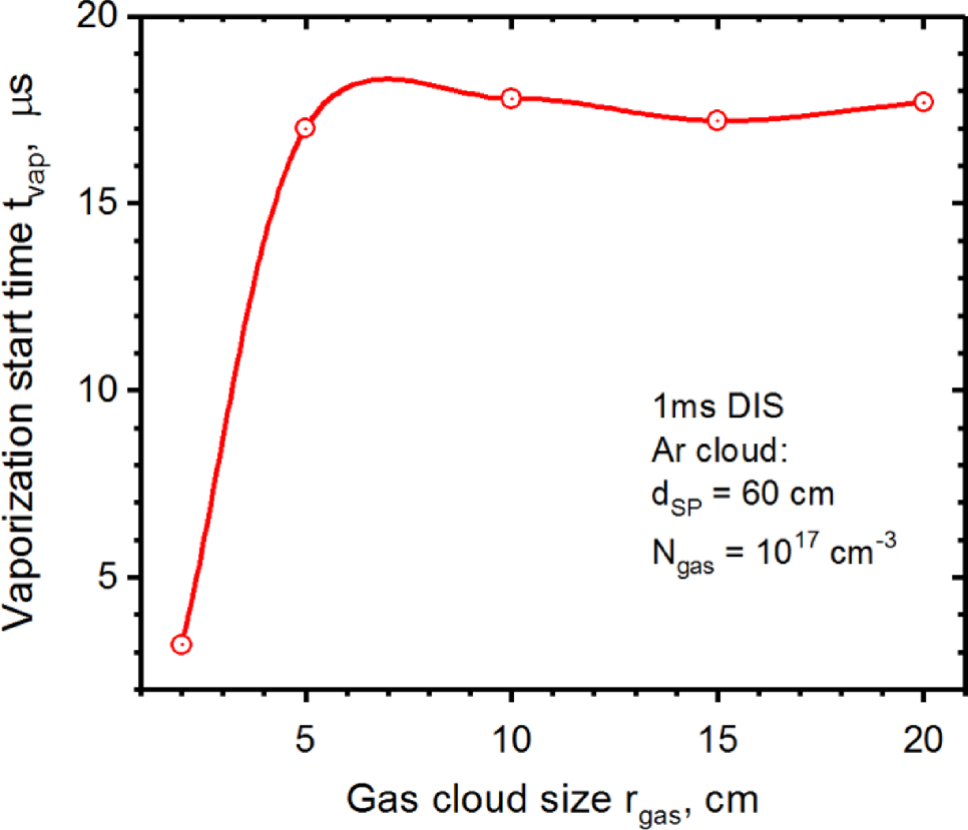
HEIGHTS simulation of 1 ms disruption with Ar cloud size variation. The images were prepared using OriginPro V2020.

As shown in Fig. 8, increasing Ar cloud size located in the middle of the separatrix also do not provide full disruption mitigation. Moreover, the vaporization spot appears in ~ 17.5 µs on the outer Baffle surface independent on the cloud size after $\:{r}_{gas}$ = 5 cm, i.e., aliening with the footprint of the disruption on the divertor plate of 10 cm wide.

**Fig. 9:**
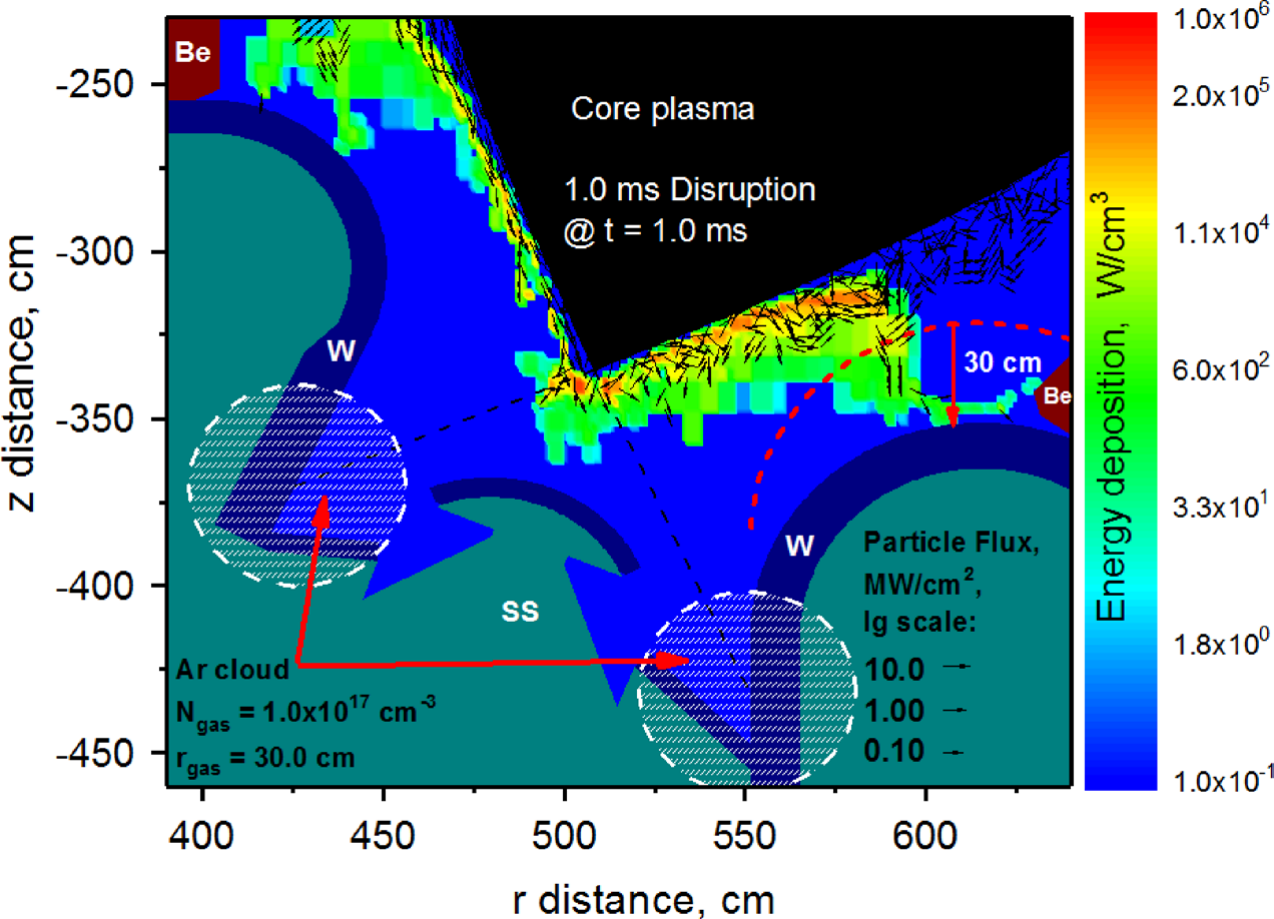
HEIGHTS simulation of 1 ms disruption with 30 cm down shifted outer Baffle surface. Red dashed line is the original ITER design. (See Supplementary Videos S1 and S2). The images were prepared using OriginPro V2020.

Based on HEIGHTS simulation results, either full airbag-like or pellet-like injection methods are not very effective for the full mitigation of the disruption event. The main issue is that the core plasma particles scatter on the dense Ar plasma cloud propagating along the SOL and deposit their energy onto the surface spot areas of the nearby components for a sufficient time to cause damage. The deposition of the scattered particles have enough time to cause Baffle vaporization for the two studied methods of mitigation. The Ar plasma cloud propagates along the divertor and Baffle surfaces but too slow to cover or protect the surface before the scattered particles impact. To overcome these issues and explore other possible mitigation methods, we investigated a possible slight variation in Baffle design and implemented a parameter into HEIGHTS simulation for a vertical surface shift either up or down but keeping everything else the same, which may not be possible for the current ITER design. We simulated ITER disruption event with the Baffle component down shifted up to − 40 cm and predicted a full mitigation at the − 30 cm Baffle surface shift. Figure 9 shows the calculated particles fluxes and energy deposition at the end of ITER disruption without any surface vaporization. (See Supplementary Video S1 for HEIGHTS dynamic simulations of the Baffle surface temperature for the modified design).

The red dashed line represents the original ITER design of the Baffle surface. For this mitigation simulation, we assumed full divertor plates cover with Ar gas cloud size $\:{r}_{gas}$ = 30 cm and distance from SP $\:{d}_{SP}$ = 1 cm. The injected Ar gas density was taken as $\:{N}_{gas}$ = 1.0 × 10^17^ cm^−3^. (See Supplementary Video S2 for HEIGHTS dynamic simulation of impact fluxes in divertor space during 1 ms transient event).

**Fig. 10:**
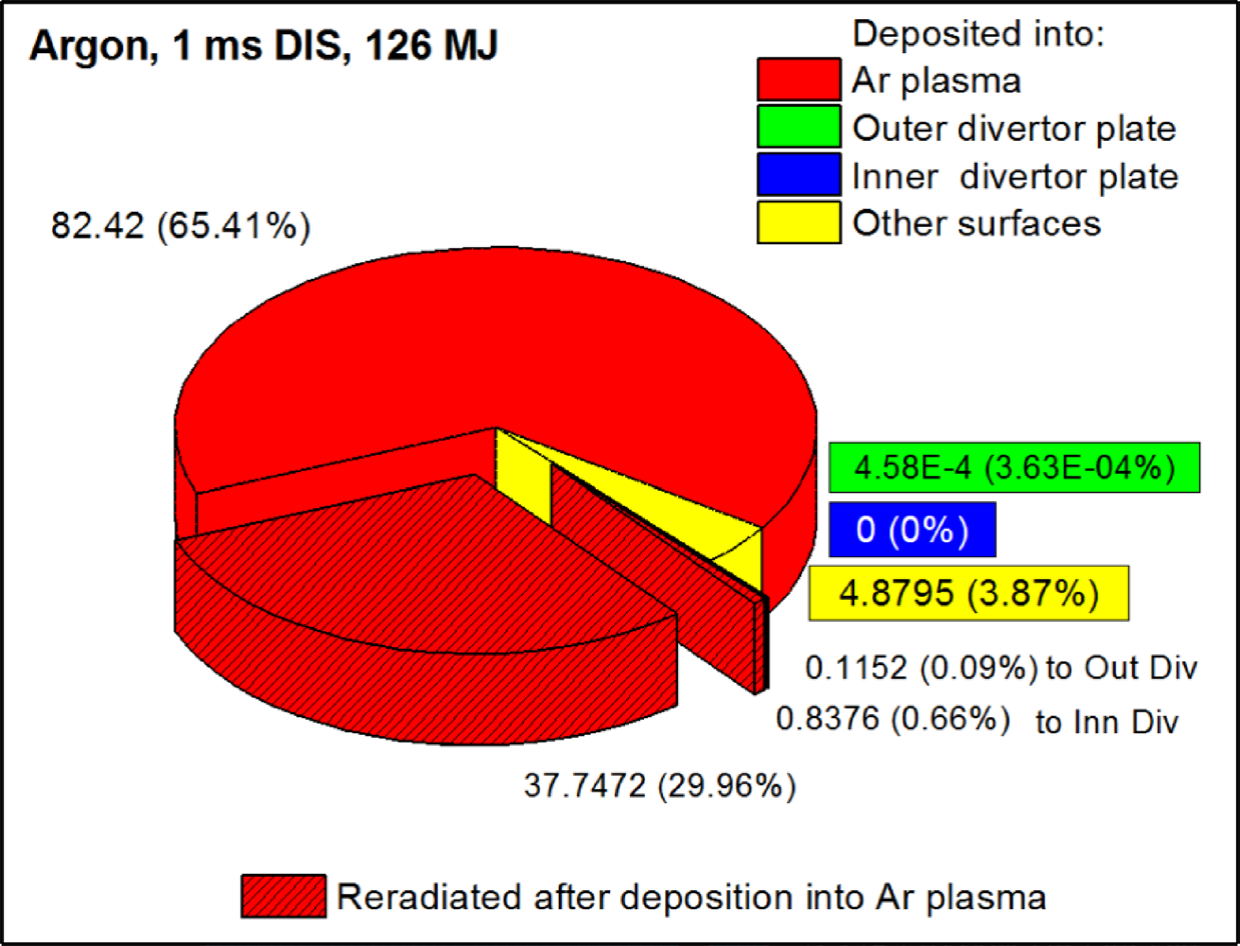
HEIGHTS simulation of 1 ms disruption with the Ar cloud size variation. The images were prepared using OriginPro V2020.

Figure 10 shows the energy balance for this fully mitigated disruption event. The red sectors show the energy deposited into Ar gas cloud that represented 96.1% of the total disrupted plasma energy. The divertor plates were completely shielded from the core plasma particles inlet (green and blue sectors). Only 3.87% of the disrupted energy was deposited into all other nearby surfaces directly by the core plasma particles (yellow sector). It should be noted that the large portion of the energy deposited into Ar cloud (30.7%) was reradiated as photons (hatched sectors). The inner and outer divertor plates were irradiated slightly (0.1% and 0.66% correspondingly). This is the consequence of the shielding effect in the detachment mode.

Therefore, most of the energy deposited from the disrupting plasma is either reradiated from the Ar plasma (as shown in Fig. 10) or stored in the evolving Ar plasma and eventually will be dissipated to larger areas as it cools down without significant heat effects. In this study, we are also calculating the various energy balances at the end of the 1 ms disruption event.

We summarized the results of the energy distribution for W, C and Li divertor in ITER design^8,11,12^ and the disruption mitigation by the Ar gas injection in Table 1. As expected, the energy deposition into the secondary plasma is the largest in the Ar gas injection case in comparison to the open/unshielded SP cases (W, C, Li). The divertor plates are completely shielded for the disrupted particles while for the unshielded SP cases an energy portion ~ 1% should be spent in the formation of the secondary plasma cloud. Despite the fact that large part of the deposited energy into Ar gas is reradiated (30.7%) which is comparable to the W plasma (44.27%), the divertor plates are not overheated. The photon reradiated energy that deposited into the divertor plates from the Ar plasma is below 1% similar to the unshielded SP cases.

**Table 1:** Final energy balance in ITER transient events with W, C, Li and ar secondary plasmas.

	Deposited from disrupting plasma %			Reradiated from photons %		
	Into second. plasma	Into outer plate	Into inner plate	From second. plasma	Into outer plate	Into inner plate
W	85.1	1.29	1.02	44.27	1.39	1.18
C	92.2	1.33	0.79	4.95	0.13	0.08
Li	88.2	0.41	0.25	1.26	0.05	0.02
Ar	96.1	0.00036	0	30.7	0.09	0.66

## Summary and conclusion

During unmitigated disruptions, the full energy of the pedestal plasma is deposited into the divertor plates that lead to significant damage to divertor and all internal components including the first wall. The core plasma impact vaporizes the divertor material and create a secondary high-Z dense plasma of the divertor plate material. The secondary plasma can be an efficient shield from the hot core particles. In our previous studies, we simulated tungsten, carbon, and lithium as potential SP materials and correspondingly as secondary plasmas for the detachment^8,11,12^. All simulations confirmed significant surface melting, vaporization, and erosion of the divertor (especially outer Baffle) during the disruption. The surface damage was larger with the higher Z of the secondary plasma of the divertor plate material. However, any credible tokamak design must tolerate few unmitigated transient events. In this work, we studied the potential of using neutral gas injections for a full mitigation of ITER disruption and to significantly reduce the damage with respect to unmitigated events.

The objective of this work was to study in a comprehensive way the integrated simulation of neutral gas injection into the divertor space to substantially reduce the heat load and to prevent vaporization of various interior components. The argon gas was chosen for the injection. We varied the density, size, and location of Ar gas cloud to minimize the energy deposition into the divertor components during the disruption. HEIGHTS modeling showed that gas injection methods are inefficient for full mitigation of disruption in the original ITER design and to prevent vaporization of interior components. However, the simulation predicted that full protection of the Baffle surfaces could be possible by slight design modification or shifting down the baffle design, keeping everything else the same. We realize that this option may not be easily implemented at this time. However, this analysis showed that using inert gas injection for successive ITER-like devices and future DEMO could hopefully lead, through various design optimizations, to significant enhancement in components lifetime during transient events.

## Methods

Methods details, including statements of data availability and any associated accession codes and references, are also available at 10.1038/s41598-021-81510-2, 10.1038/s41598-022-08837-2 and 10.1038/s41598-022-21866-1.

The benchmarking of HEIGHTS models can also be found in Refs^13–15,17–20^. The Ref^6^. included comparison with the UEDGE calculations and NSTX experiment. We updated our HEIGHTS radiation transport calculation in the argon plasma with a detailed consideration of energy transfer in strong lines along with the continuum spectra. To allow simulation of RT having strong lines, we optimized the initial opacity tables and separated the full plasma spectrum into spectral groups where optical coefficients are relatively invariable. Using such technique, the opacity tables were reduced by an order of magnitude for complex elements as tungsten and by two orders of magnitude for the lighter elements such as argon.

**Fig. 11:**
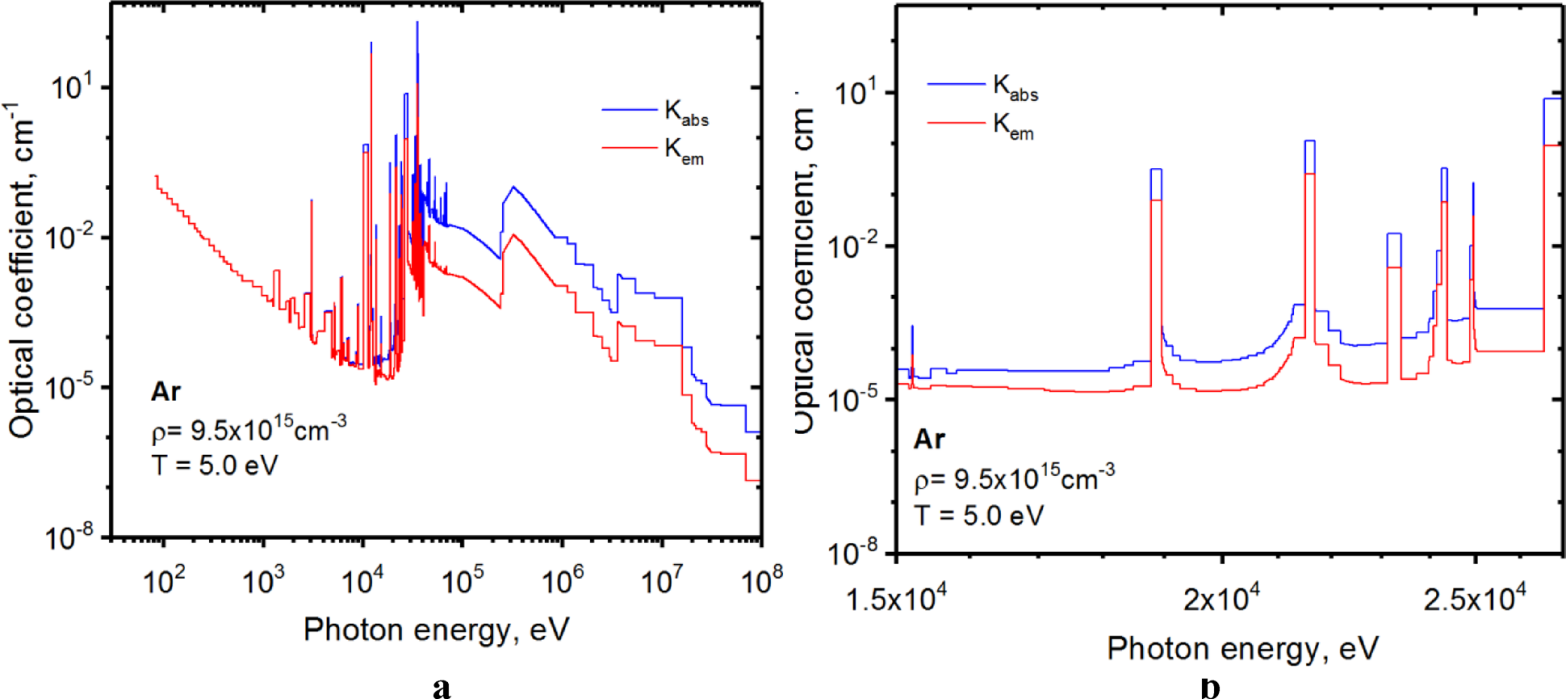
Optimized opacities of Ar plasma for RT calculations: full spectrum **a**, and fine structure **b**. The images were prepared using OriginPro V2020.

Figure 11 shows an example of optimization of Ar opacities for 5.0 eV temperature and 9.5 × 10^15^ cm^− 3^ ionic concentration. Because the plasma spectrum depends critically on the temperature, the collected spectral groups are created for the large set of temperatures. The spectrum fine structure with separation of strong lines in the area of photon energy ~ 20 keV is shown in Fig. 11b.

## Supplementary Information

Below is the link to the electronic supplementary material.

Supplementary Material 1

Supplementary Material 2

Supplementary Material 3

## Data Availability

The data that support the findings of this study are stored on Purdue Servers and on Argonne National Laboratory Bebop cluster and are available from the corresponding authors upon reasonable request.

## Declarations

Correspondence and requests for materials should be addressed to V.S.
