# Supplementary material for: Comprehensive analysis of disruption mitigation methods using gas and pellet-like injections in ITER-like Tokamaks
Source: Sci Rep. 2025 Sep 22;15:32630. doi: 10.1038/s41598-025-14407-z (PMC12454634; doi:10.1038/s41598-025-14407-z)
Supplement: Supplementary file 3 — Supplementary Material 3 [file 41598_2025_14407_MOESM3_ESM.docx]

Supplementary video legends

**Supplementary Video S1.** HEIGHTS dynamic simulations of the Baffle surface temperature for the modified design.

**Supplementary Video S2.** HEIGHTS dynamic simulation of impact fluxes in divertor space during 1 ms transient event.
